# In vitro analysis of the effect of Flightless I on murine tenocyte cellular functions

**DOI:** 10.1186/s13018-020-01692-9

**Published:** 2020-05-12

**Authors:** Jessica E. Jackson, Zlatko Kopecki, Peter J. Anderson, Allison J. Cowin

**Affiliations:** 1 Regenerative Medicine, Future Industries Institute, University of South Australia, Adelaide, South Australia Australia; 2 Faculty of Medicine and Health, University of Adelaide, Adelaide, South Australia Australia

**Keywords:** Flii, Flightless I, Tendon, Healing

## Abstract

**Background:**

Healing of tendons after injury involves the proliferation of tenocytes and the production of extracellular matrix; however, their capacity to heal is limited by poor cell density and limited growth factor activity. Flightless I (Flii) has previously been identified as an important regulator of cellular proliferation and migration, and the purpose of this study was to evaluate the effect of differential Flii gene expression on tenocyte function in vitro.

**Methods:**

The role of Flii on tenocyte proliferation, migration, and contraction was assessed using established assays. Tenocytes from *Flii*^*+/−*^, wild-type, and *Flii* overexpressing mice were obtained and the effect of differential *Flii* expression on migration, proliferation, contraction, and collagen synthesis determined in vitro*.* Statistical differences were determined using unpaired Student’s *t* test and statistical outliers were identified using the Grubbs’ test.

**Results:**

*Flii* overexpressing tenocytes showed significantly improved migration and proliferation as well as increased collagen I secretion. Explanted tendons from *Flii* overexpressing mice also showed significantly elevated tenocyte outgrowth compared to *Flii*^*+/−*^ mice. In contrast to its role in dermal wound repair, Flii positively affects cellular processes in tendons.

**Conclusions:**

These findings suggest that Flii could be a novel target for modulating tenocyte activity and improving tendon repair. This could have significant clinical implications as novel therapeutic targets for improved healing of tendon injuries are urgently needed.

## Background

Tendons inherently have minimal capacity for healing owing to poor cell density and growth factor activity [1]. Healing of tendons involves the proliferation of spindle-shaped fibroblast-like cells known as tenocytes, which comprise the main cellular content of mature tendons. Tenocytes have long cellular processes which facilitate cell-extracellular matrix (ECM) and cell-cell communication through desmosomes and tight junctions [2]. They are highly metabolically active and are responsible for synthesizing and secreting ECM. Tendon repair is characterized by the influx of intrinsic and extrinsic tenocytes which migrate to the injured area and synthesize and secrete ECM to begin the repair process [3]. Tendon healing can occur solely through epitenon-based intrinsic tenocytes that secrete larger, more mature collagen fibers compared with extrinsic tenocytes, resulting in improved physiological outcomes [4]. Tenocyte characterization during injury repair is poorly understood; however, it is well established that tenocyte function is to secrete ECM components and growth factors during healing [5]. Of particular importance is their secretion of transforming growth factor beta (TGFβ) ligands as this family of growth factors is active throughout all phases of tendon repair [5–7].

Tenocyte migration, proliferation, and contraction are essential processes that allow tendon healing to occur. The actin cytoskeleton is the driving force behind these processes and it also has essential roles in transmembrane signaling and healing [8, 9]. In response to injury, the actin cytoskeleton is remodeled to facilitate the permeation of inflammatory cells and promote tenocyte migration [4, 10]. Flightless (Flii) is a highly conserved actin remodeling protein that contains both a gelsolin and leucine-rich repeat (LRR) domain enabling it to modulate the cytoskeleton by regulating actin polymerization and depolymerization [11–14]. Flii is a known regulator of cellular migration, proliferation, contraction, and adhesion; however, this has been mainly characterized in cutaneous healing responses [8, 15–17]. Flii is expressed ubiquitously throughout cells and tissues of the body but its specific expression in cells and structures with migratory capabilities is of particular interest due to their involvement with wound repair [18, 19]. Flii has previously been determined to be an important negative regulator of cutaneous wound healing and skin blistering [8, 14, 16–18]. There is also strong evidence to suggest that Flii modulates TGFβ expression, with decreased levels of TGFβ1 detected in cutaneous healing studies in mice with low levels of Flii (*Flii*^*+/−*^) [20]. In addition, reducing the levels of Flii decreases pro-scarring TGF-β1 and Smad 3, while causing an elevation of anti-scarring inhibitory Smad 7, suggesting a mechanistic role for Flii in TGF-β signaling [16, 20].

In this study, the effect of altering *Flii* expression on tenocyte activity was investigated. Primary tenocytes were extracted from digital tendons of *Flii*^*+/−*^*,* wild-type (WT), and *Flii*^*Tg/Tg*^ mice and the effect of differential *Flii* expression on their migration, proliferation, contraction, and ECM production assessed.

## Methods

### *Flii* mouse generation

All mouse strains were congenic on the Balb/c background and Balb/c littermates were used as wild-type (*WT*) control animals. *Flii* homozygous knockout mice are embryonic lethal due to defects with gastrulation during early embryogenesis [21]. *Flii* heterozygous mice (*Flii*^*+/−*^) are phenotypically normal, fertile with normal life expectancy. Mice carrying the complete human *Flii* gene on a cosmid transgene were maintained as described previously [8, 21]. Heterozygous transgenic mice *Flii*^*Tg/+*^ were made by crossing *Flii*^*+/+*^ with cosmid transgene *Flii*^*+/−*^. These transgenic mice were inter-crossed to obtain animals homozygous for the transgene *Flii*^*Tg/Tg*^ and carry two copies of the mouse *Flii* gene and two additional copies of the human *Flii* gene (*Flii*^*Tg/Tg*^) resulting in elevated levels of Flii protein in various tissues [8, 19].

### Isolation of mouse tenocytes

**Fig. 1:**
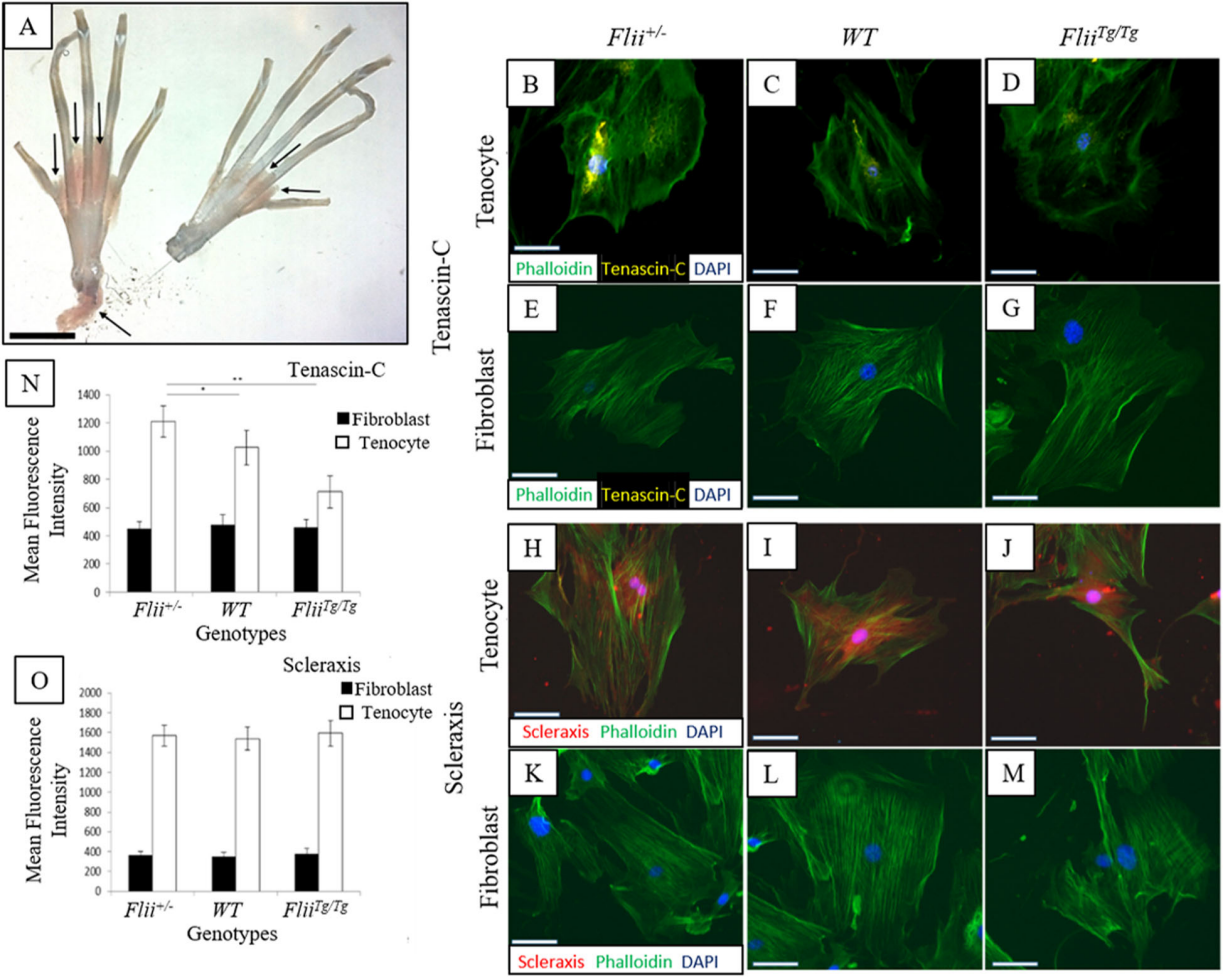
Tenascin-C and Scleraxis are expressed specifically in tenocytes. **a** Intact digital tendons were removed from the right and left hind paws of mice for tenocyte isolation. The tendon sheath is indicated by arrows and was removed before digestion to ensure a pure population of intrinsic tenocytes. Scale bar = 1 cm. Representative images of Tenascin C (**b**–**g**) and Scleraxis (**h**–**m**) staining in tenocyte and fibroblast cells isolated from *Flii*^*+/−*^, WT and *Flii*^*Tg/Tg*^ mice. Positive staining was detected in tenocyte cells for both Tenascin-C and Scleraxis, and no expression was noted in fibroblasts. Magnification × 20, scale bar = 100 μM. Graphical representation of mean fluorescence intensity of Tenascin C (**n**) and Scleraxis (**o**) staining. Tenascin-C staining was significantly higher in *Flii*^*+/−*^ cells compared to WT and *Flii*^*Tg/Tg*^ cells. Data is represented as mean ± SEM. *p ≤ 0.05, **p ≤ 0.01, *n* = 6

Murine tenocyte isolation can generally be achieved from adult mice (12–16 weeks) following established protocols [22]. Therefore, digital tendons were removed intact from the hind feet of 4 euthanized female 12-week-old *Flii*^*+/−*^, WT, and *Flii*^*Tg/Tg*^ mice using micro dissection (Fig. 1a). The tendon sheath was removed, and the tip and base of the tendons discarded. The remaining tendons were chopped into 1 mm^3^ sections and digested overnight in 4 mg/mL dispase (Worthington Biochem, NJ, USA), and 0.5 mg/mL collagenase (Worthington Biochem, NJ, USA) in Dulbecco’s modified Eagle’s media (DMEM) without serum at 37 °C. The following day, the tendon mix was put through a 70-μM cell strainer (In Vitro Technologies, Victoria, Australia) and the remaining tissue discarded. The tube and strainer were washed with sterile media (DMEM + 10% fetal bovine serum [FBS] + penicillin/streptomycin [Pen/Strep] + fungizone) and excess media added to stop the action of the dispase/collagenase mix. The suspension was spun at 1200 rpm for 5 min and the supernatant discarded. The pellet was resuspended in 4 mL sterile media and plated on collagen-coated T25 flask. The T25 was left in an incubator (37 °C, 5% CO_2_) for approximately 7 days with media changes every 2–3 days until the cells were a minimum of 90% confluent [23, 24].

### Migration assay

Tenocytes isolated from *Flii*^*+/−*^, WT, and *Flii*^*Tg/Tg*^ mice were plated into 96-well plates at 5 × 10^5^ cells/mL and left overnight in an incubator at 37 °C at 5% CO_2_ to reach confluence. A Woundmaker™ (Essen Bioscience, Michigan, USA) was used to create uniform wounds of 7–800 μM in each well of the 96-well plate which was subsequently placed into an Incucyte (Essen Bioscience, Michigan, USA) at 37 °C and 5% CO_2_ where images were automatically taken every 3 h for 24 h. The resulting images were analyzed using Image Pro Plus 7.1 as previously described [25].

### Tendon outgrowth assay

Whole tendons were removed in sterile conditions from the hind feet of 6 *Flii*^*+/−*^, WT, and *Flii*^*Tg/Tg*^ mice. Five millimeters was removed from the ends of each tendon and the remaining tendon was cut into 3 mm^3^ sections, transferred into 12-well plates (1 section per well), and cultured in DMEM + 20% FBS + Pen/Strep + Fungizone. Cultures were maintained at 37 °C at 5% CO_2_ for 12 days [26]. Images were taken at days 0, 4, 8, and 12. Migration distances were measured every 90° using Image Pro Plus 7.1 (MediaCybernetics Inc., Maryland, USA) and the average distance calculated.

### Collagen immunoassay

A collagen immunoassay was employed similar to previous studies [27]. Briefly isolated *Flii*^*+/−*^, WT, and *Flii*^*Tg/Tg*^ tenocytes were seeded into 96-well plates at 5 × 10^5^ cells/mL in media (DMEM + 20% FBS + Pen/Strep + Fungizone) and left overnight at 37 °C 5% CO_2._ Following serum starvation, media were replaced with serum-free media plus Pen/Strep and Fungizone and left for 60 h to allow collagen accumulation. The cells were subsequently washed 3 times in phosphate-buffered saline (PBS), then fixed and permeabilized in ice-cold methanol for 15 min at − 20 °C. The cells were aspirated and briefly air-dried before being washed 3 times in PBS. Cells were treated with 0.5% Tween 20 in PBS for 10 min and blocked in PBS + 3% normal goat serum for 30 min. The cells were incubated in 50 μL per well collagen I (#600-401-103; Rockland, PA; 2 μg/mL) in PBS and 3% normal goat serum for 2 h at room temperature. The cells were washed and incubated in 50 μL per well secondary antibody in PBS for 1 h in the dark at room temperature. Once the secondary staining was complete, the cells were washed in PBS, stained with 4′,6-diamindino-2-phenylindole (DAPI) at 1:5000 for 1 min and analyzed immediately on an Olympus Epifluorescent microscope as previously described [13].

### Collagen contraction assay

Primary tenocytes and fibroblasts isolated from *Flii*^*+/−*^, WT, and *Flii*^*Tg/Tg*^ mice tendons or skin, respectively, were trypsinized in 10 × trypsin/EDTA (Sigma-Aldrich, Sydney, Australia) for 5 min at 37 °C and quenched using DMEM + 20% FBS. The cells were spun for 5 min at 1200 rpm and resuspended in ice-cold DMEM at 1 × 10^6^ cells/mL. 3D collagen gels were prepared by mixing 8 parts of chilled collagen solution (2 mg/mL) with 1 part 10 × DMEM containing 10% FBS as previously described [16]. The pH was adjusted to 7.4 using 0.1 M NaOH. The tenocytes and fibroblasts were added to the collagen gel mixture at 1 × 10^5^ cells/mL. Five hundred microliters of this mixture was added per well to a flat-bottomed 48-well plate and allowed to set for 120 min at 37 °C 5% CO_2_. Following this, the gel was carefully dislodged using a 200-μL pipette and the gels floated by the addition of 1 mL DMEM (+ 20% FCS, Pen/Strep, and Fungizone). Images were taken at 24, 48, and 72 h post media addition using Olympus Epifluorescent microscope and the images analyzed for degree of contraction using Image Pro-Plus 7.1 as previously described [16].

### Immunocytochemistry

Immunocytochemistry was undertaken as previously described [13]. Briefly, isolated *Flii*^*+/−*^, WT, and *Flii*^*Tg/Tg*^ tenocyte cells were plated onto glass coverslips and fixed and stained for Flii (2 μg/mL; sc-21716, Santa Cruz Biotechnology, CA), TGBβ1(2 μg/mL; sc-52893 Santa Cruz Biotechnology CA), β-tubulin (2 μg/mL, T4026, Sigma-Aldrich, NSW), collagen I (2 μg/mL #600-401-103, Rockland, PA), Tenascin-C (2 μg/mL, sc-25328 Santa Cruz Biotechnology CA), and Scleraxis (2 μg/mL, sc-87425 Santa Cruz Biotechnology CA). Cells were subsequently stained with phalloidin-FITC (2 μg/mL #P5282-0.1G, Sigma-Aldrich, NSW) and nuclear counterstain 4,6-diamidino-2-phenyindole DAPI (2 μg/mL D9564, Sigma-Aldrich, NSW). The cells were mounted in fluorescent mounting media (DAKO, CA) and viewed under an Olympus Epifluorescent microscope. Average stain-specific fluorescence was measured using AnalySiS (Soft-Imaging System GmbH, Munster, Germany).

### WST-1 proliferation assay

Methods were as previously described [25]. Briefly, cells were serum-starved for 6 h to synchronize the cell cycle. The media was then replaced with DMEM + 20% FBS and incubated at 37 °C, 5% CO_2_ for 24, 48, or 72 h. Ten microliters WST-1 was added to each well and the absorbance read on a microplate reader at 450 nm and 600 nm. Cell proliferation was normalized relative to cells with no serum added as control.

### Statistical analysis

Statistical differences were determined following unblinding of the data using GraphPad software (USA) using the unpaired Student’s *t* test. A *p* value of less than 0.05 was considered statistically significant. A *p* value of less than 0.01 was considered highly significant. Statistical outliers were determined using the Grubbs’ test with a significance level of 0.05.

## Results

### Tenocytes express Tenascin-C and Scleraxis

Digital tendons were removed from *Flii*^*+/−*^, WT, and *Flii*^*Tg/Tg*^ mice, the tendon sheath was removed by microscopic dissection, and intrinsic tenocytes were isolated by outgrowth or enzyme digestion methods (Fig. 1). Specific care was taken to remove the ends of the tendons to avoid phenotypically different tenocytes at the myotendinous junction (MTJ) and osteotendinous junction (OTJ). Dermal fibroblasts were also isolated from *Flii*^*+/−*^, WT, and *Flii*^*Tg/Tg*^ by outgrowth or enzyme digestion methods. Tenocytes are structurally similar to fibroblasts and it is impossible to visually distinguish the two cell-types. Tendon-specific markers Tenascin-C and Scleraxis were used to identify tenocytes (Fig. 1b–d, h–j). Tenascin-C is a glycoprotein expressed specifically in tenocytes [28]. Scleraxis is uniquely expressed in tissues that form tendons and ligaments [29]. Tenascin-C and Scleraxis were expressed in *Flii*^*+/−*^, WT, and *Flii*^*Tg/Tg*^ tenocytes with no expression seen in fibroblasts from the same mice, (Fig. 1b–g). Tenascin-C staining was observed to be peri-nuclear whereas Scleraxis staining was observed in the cytoplasm as well as nuclear regions (Fig. 1b–g, h–m). Tenascin-C was significantly higher in *Flii*^*+/−*^ tenocytes when compared to WT and *Flii*^*Tg/Tg*^ (*p* = 0.042 and 0.008, respectively) but no significant difference was noted in Scleraxis expression in tenocytes across the three *Flii* genotypes (Fig. 1n, o).

### Flii is differentially expressed in *Flii*^*+/−*^, WT, and *Flii*^*Tg/Tg*^ tenocytes in response to scratch wounding

**Fig. 2:**
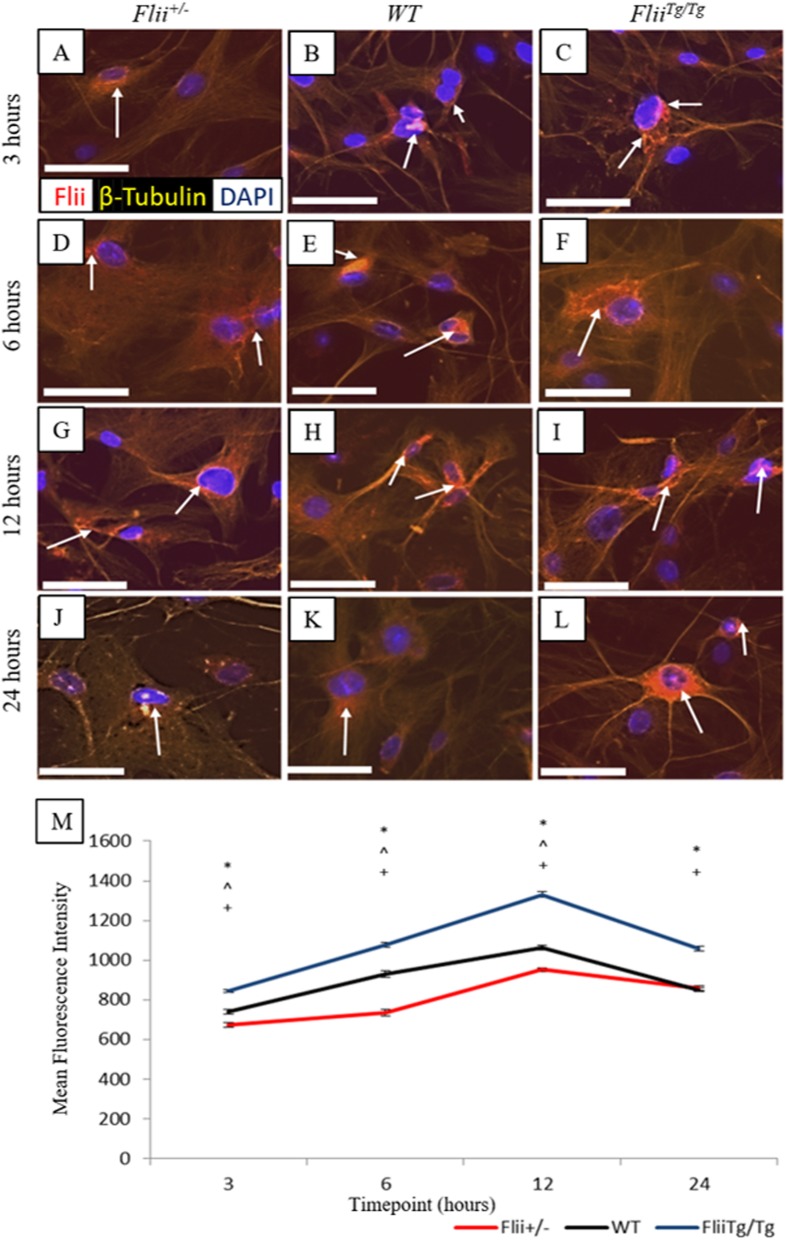
Flii expression in *Flii*^*+/−*^, WT and *Flii*^*Tg/Tg*^ tenocytes. Confluent tenocytes were scratch-wounded and stained for Flii expression at 3, 6, 12, and 24 h post-wounding. **a**–**l** Representative images showing Flii expression in *Flii*^*+/−*^, WT, and *Flii*^*Tg/Tg*^ tenocytes at 3, 6, 12, and 24 h post-wounding. Magnification × 10. Scale bar = 100 μM. Flii represented by white arrows (**m**) Graphical representation of Flii expression in tenocytes. *Flii*^*Tg/Tg*^ tenocytes express significantly more Flii than WT and *Flii*^*+/−*^ tenocytes. *p ≤ 0.05 between *Flii*^*+/−*^ and *Flii*^*Tg/Tg*^. ^^^*p* ≤ 0.05 between *Flii*^*+/−*^ and WT. ^+^*p* ≤ 0.05 between *Flii*^*Tg/Tg*^ and WT. Data represented as mean ± SEM. *n* = 6

Previous studies have shown that Flii is upregulated in fibroblasts in response to wounding [9]. Here we examined Flii levels in wounded *Flii*^*+/−*^, WT, and *Flii*^*Tg/Tg*^ tenocytes. Confluent monolayers of tenocytes isolated from *Flii*^*+/−*^, WT, and *Flii*^*Tg/Tg*^ mice were scratch wounded and immunofluorescently stained for Flii at 3, 6, 12, and 24 h post-wounding. Representative images can be seen in Fig. 2a–l. *Flii*^*Tg/Tg*^ tenocytes showed significantly higher Flii expression at 3, 6, 12, and 24 h than WT (*p* = 0.0006, 0.0002, 0.00002, and 0.00002, respectively) and *Flii*^*+/−*^ tenocytes (*p* = 0.000005, 0.00005, 0.00001, and 0.00003, respectively) (Fig. 2a–m). Flii staining was apparent at all time points and peaked at 12 h post-wounding. The staining was mainly perinuclear in distribution, although some cytoplasmic staining was also seen in the later time points.

### Tenocyte migration is regulated by Flii

**Fig. 3:**
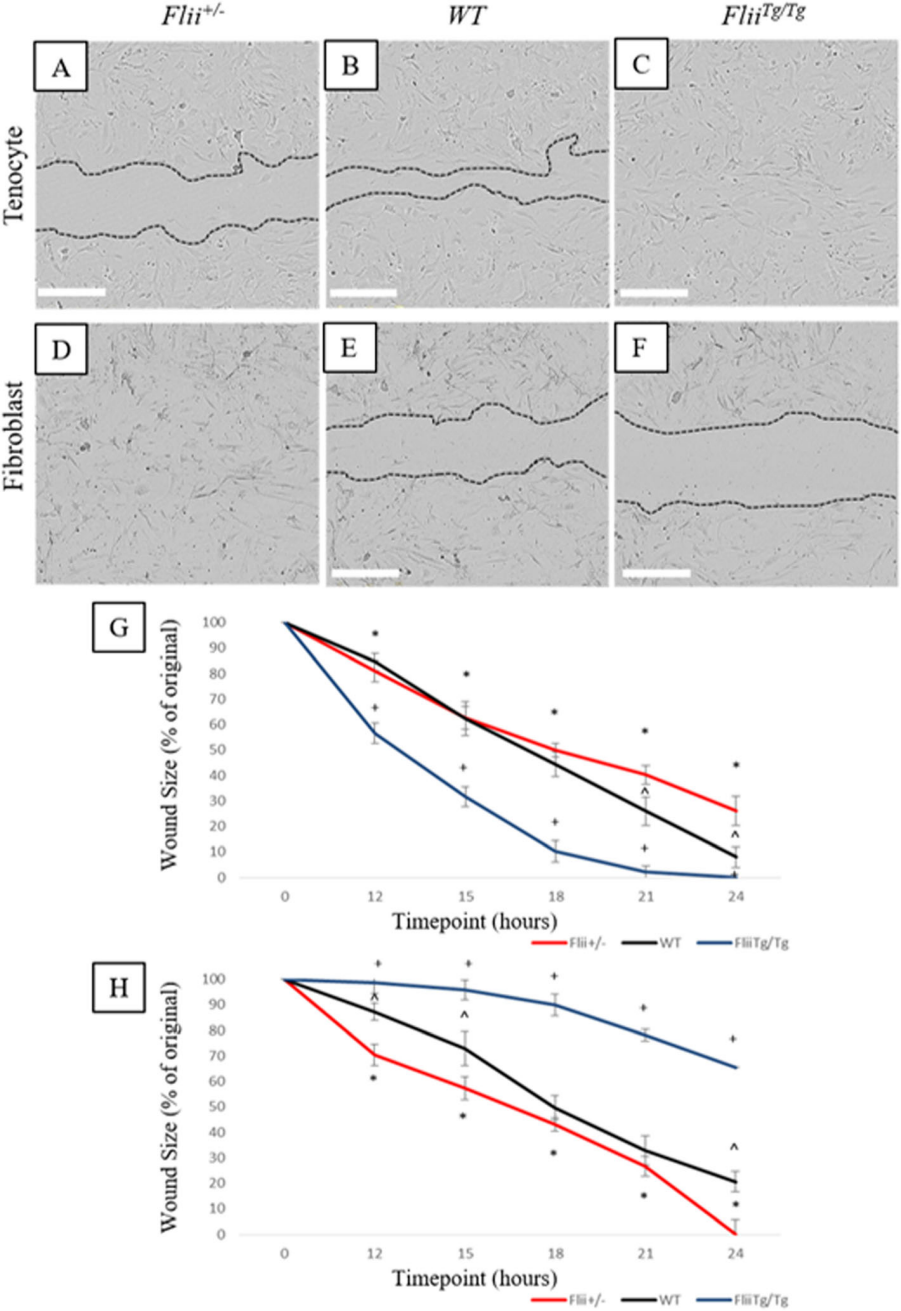
*Flii* regulates tenocyte cellular migration. An in vitro scratch wound model was used to investigate the effect of differential *Flii* on tenocyte migration. **a**–**c** Representative images of scratch wounds in *Flii*^*+/−*^, WT, and *Flii*^*Tg/Tg*^ tenocytes at 24 h post-wounding. **d**–**f** Representative images of scratch wounds in *Flii*^*+/−*^, WT, and *Flii*^*Tg/Tg*^ fibroblasts at 24 h post-wounding. Magnification × 4. Scale bar = 300 μM. **g** Graph showing scratch closure in tenocytes expressed as percentage of original wound size. *Flii*^*Tg/Tg*^ tenocytes show significantly improved wound closure. **h** Graph showing scratch closure in fibroblasts expressed as percentage of original wound size. *Flii*^*+/−*^ fibroblasts show significantly improved wound closure. *p ≤ 0.05 between *Flii*^*+/−*^ and *Flii*^*Tg/Tg*^. ^^^*p* ≤ 0.05 between *Flii*^*+/−*^ and WT. ^+^*p* ≤ 0.05 between *Flii*^*Tg/Tg*^ and WT. Data represented as mean ± SEM. *n* = 6

Previous studies have established the role for Flii in regulating cell migration [9, 15]. Here we examined the effect of Flii on tenocyte migration and compared it to effects seen in fibroblasts. Isolated primary tenocytes and fibroblasts from *Flii*^*+/−*^, WT, and *Flii*^*Tg/Tg*^ mice were scratch wounded and cellular migration measured over 24 h. Increased *Flii* expression resulted in faster cellular migration in tenocytes (Fig. 3a–c) with the *Flii*^*Tg/Tg*^ tenocytes showing significantly faster migration than *Flii*^*+/−*^ tenocytes across the entire time course measured (*p* ≤ 0.001) (Fig. 3g). *Flii*^*Tg/Tg*^ tenocytes also showed significantly faster migration than WT tenocytes at 21 and 24 h post-scratch wounding (*p* = 0.05 and 0.02, respectively). At 24 h post-scratch wounding, *Flii*^*Tg/Tg*^ tenocyte wounds had fully closed whereas *Flii*^*+/−*^ tenocyte wounds were still 25% open, suggesting that increased levels of Flii improve the migratory ability of primary murine tenocytes. In contrast, murine fibroblast cells showed that decreased *Flii* expression improved cellular migration (Fig. 3d–f), with scratch wounded *Flii*^*+/−*^ fibroblasts showing significantly faster closure than *Flii*^*Tg/Tg*^ fibroblasts across the whole time course (*p* = ≤ 0.001) (Fig. 3d–f, h).

### Tenocyte cellular outgrowth is regulated by Flii

**Fig. 4:**
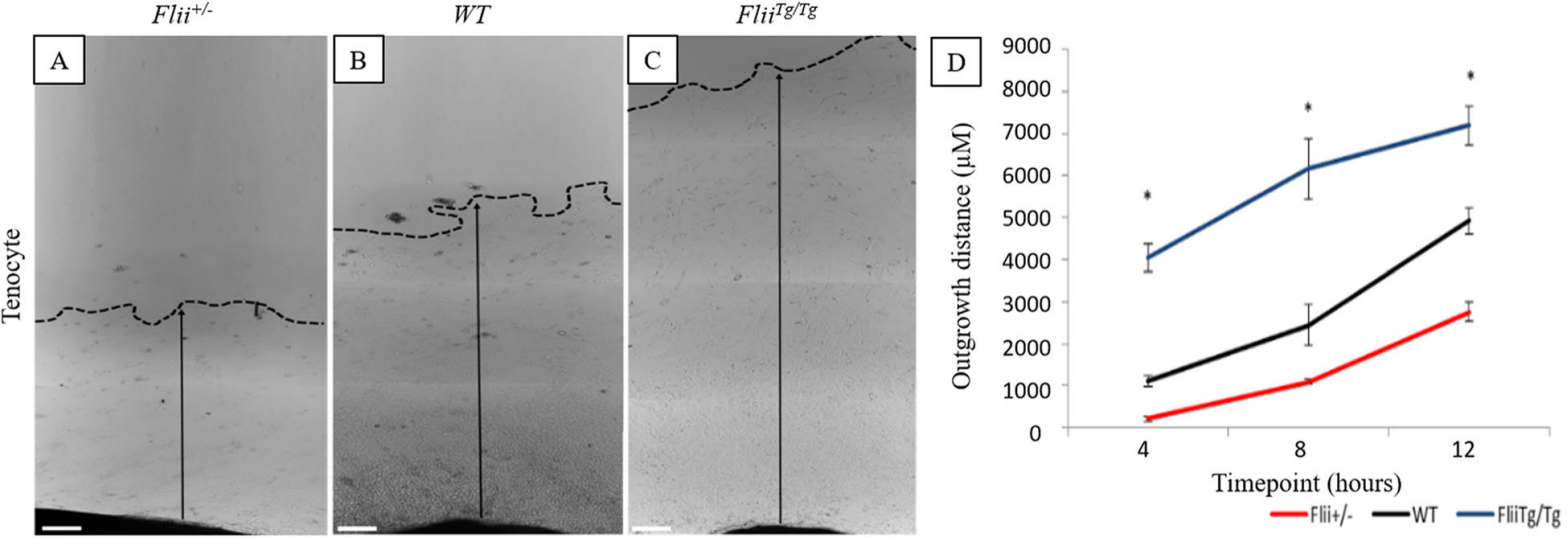
Differential effect of *Flii* expression on tenocyte outgrowth from tendon explants. 2 mm^3^ sections of tendons from *Flii*^*+/−*^, WT, and *Flii*^*Tg/Tg*^mice were cultured for 12 days and tenocyte outgrowth measured as average migration distance. **a**–**c** Representative images of cellular outgrowth in *Flii*^*+/−*^, WT, and *Flii*^*Tg/Tg*^ tenocytes at 12 days post seeding. Dotted line + arrow = outgrowth distance. Magnification × 4. Scale bar = 200 μM. **d***Flii*^*Tg/Tg*^ tenocytes have significantly increased outgrowth compared to WT and *Flii*^*+/−*^ tenocytes. Data represented as mean ± SEM. *p ≤ 0.05, *n* = 6

Flii has previously been shown to regulate cellular outgrowth including both fibroblasts and keratinocytes [9, 15]. To investigate the effect of *Flii* on tenocyte cellular outgrowth, 2 mm^3^ sections of digital tendons, cleaned of their sheaths, from *Flii*^*+/−*^, WT, and *Flii*^*Tg/Tg*^ mice were placed onto collagen type I coated plates in culture, and the average outgrowth measured over 12 days (Fig. 4a–d). *Flii*^*Tg/Tg*^ tenocytes had significantly increased cellular outgrowth across all 12 days when compared to WT and *Flii*^*+/−*^ tenocytes (*p* = 0.007 and 0.0005, respectively, at day 12), with *Flii*^*Tg/Tg*^ cells showing a 2-fold increase in cellular migration by day 12 compared to *Flii*^*+/−*^ cells (Fig. 4a–d). A dense collection of cells migrated out of *Flii*^*Tg/Tg*^ and WT explants by day 3, whereas *Flii*^*+/−*^ showed a less dense, slower initial migration. These cells stained positive for Scleraxis confirming the tenocyte phenotype (data not shown).

### *Flii*^*Tg/Tg*^ tenocytes have significantly improved proliferation

**Fig. 5:**
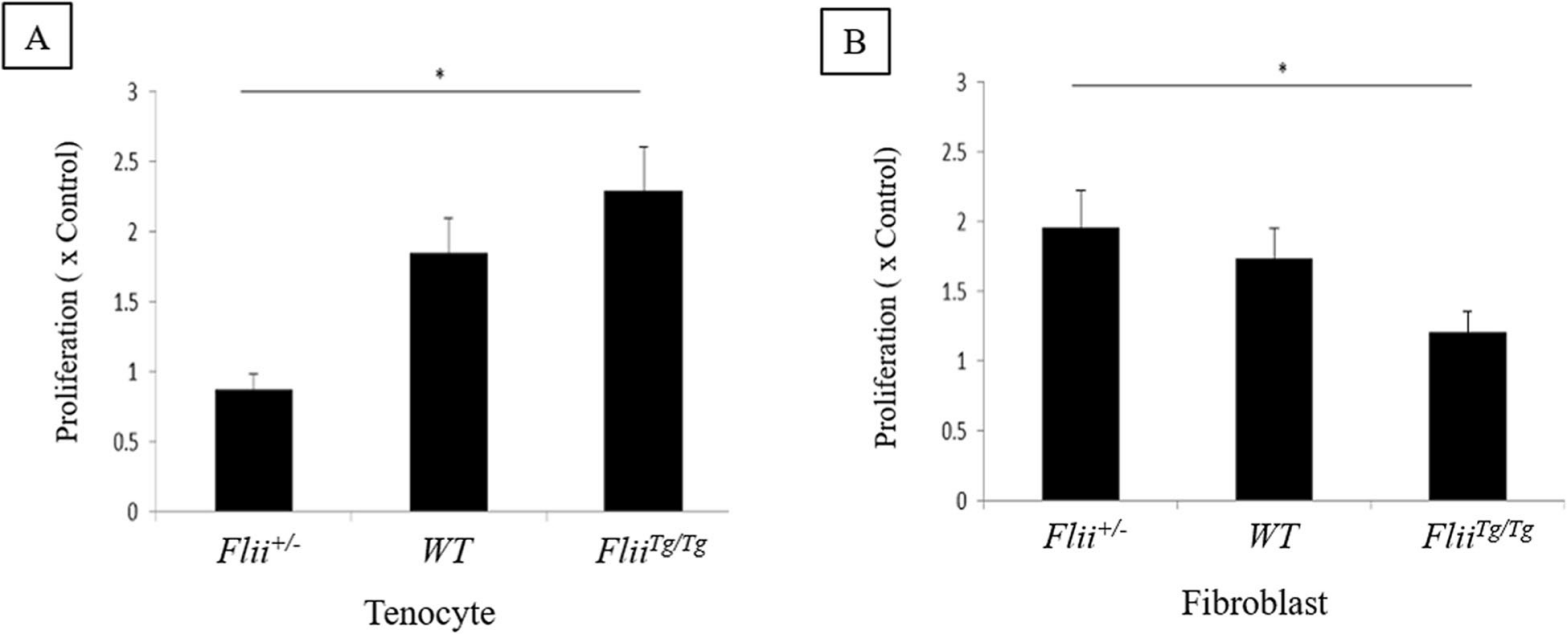
Increased Flii levels increases tenocyte proliferation. Murine tenocytes and fibroblasts from in *Flii*^*+/−*^, WT, and *Flii*^*Tg/Tg*^ mice were used in a 24-h WST-1 assay to determine the effect of Flii on cellular proliferation. **a** Graphical representation of tenocyte proliferation in the three genotypes. *Flii*^*Tg/Tg*^ tenocytes showed significantly improved cellular proliferation. **b** Graphical representation of fibroblast proliferation in the three genotypes. *Flii*^*+/−*^ tenocytes showed significantly improved cellular proliferation. *p ≤ 0.05. Data represented as mean ± SEM. *n* = 6

Using primary murine tenocytes and fibroblasts from *Flii*^*+/−*^, WT, and *Flii*^*Tg/Tg*^ mice, we examined the effect of altering the level of *Flii* on proliferation using a 24-h WST-1 assay. In tenocytes, increased *Flii* resulted in improved cellular proliferation (*p* = 0.01 compared to *Flii*^*+/−*^) (Fig. 5a). In contrast, fibroblasts showed that increased *Flii* levels resulted in a decrease in cellular proliferation (*p* = 0.03 compared to *Flii*^*+/−*^) (Fig. 5b).

### Flii has minimal effect on tenocyte contraction

**Fig. 6:**
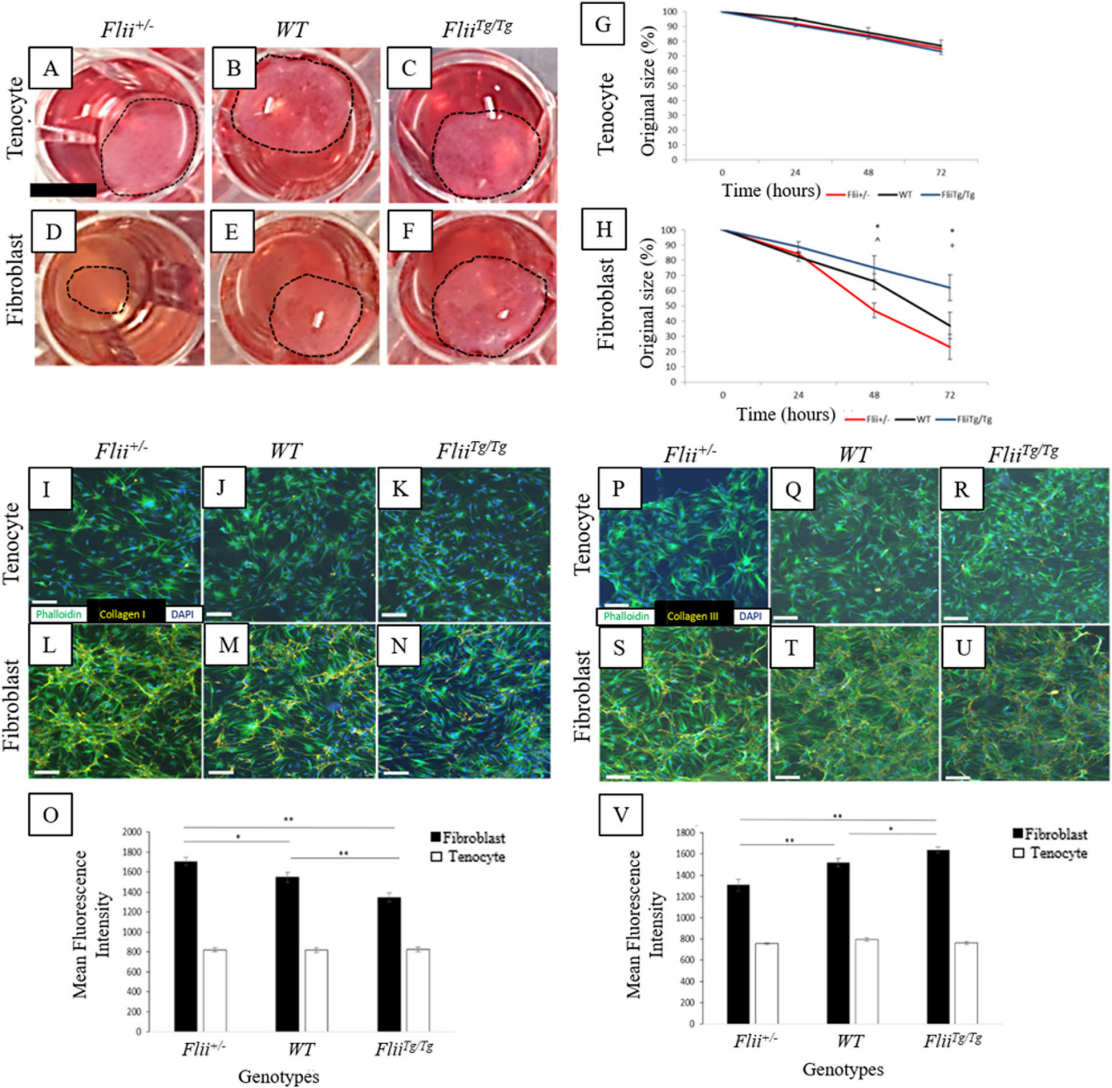
Effect of *Flii* on collagen contraction and production in fibroblasts and tenocytes. Contraction of tenocyte and fibroblast populated collagen gels was analyzed over 72 h. **a**–**c** Representative images of tenocyte populated 3D collagen gels at 72 h post-plating. **d**–**f** Representative images of fibroblast populated 3D collagen gels at 72 h post-plating. Scale bar = 2.5 mm and refers to all images. **g** No significant difference seen in contraction in tenocytes across 72 h. **h***Flii*^*+/−*^ fibroblasts have increased cellular contraction over 72 h. *p ≤ 0.05 between *Flii*^*+/−*^ and *Flii*^*Tg/Tg*^. ^^^*p* ≤ 0.05 between *Flii*^*+/−*^ and WT. ^+^*p* ≤ 0.05 between *Flii*^*Tg/Tg*^ and WT. Data represented as mean ± SEM. *n* = 6. Confluent tenocytes and fibroblasts from *Flii*^*+/−*^, WT, and *Flii*^*Tg/Tg*^ mice were left for 48 h in a 2D culture to allow the accumulation of secreted collagen type I. **i**–**k** Representative images show no significant difference in collagen type I secretion in tenocytes across all three genotypes. **l**–**n** Overexpression of *Flii* significantly decreased the level of collagen type I secretion in fibroblasts. Magnification × 10, scale bar = 200 μM. **o** Graphical representation of collagen type I secretion in fibroblasts and tenocytes. Data is represented as mean ± SEM. *p ≤ 0.05, **p ≤ 0.01. *n* = 6. Confluent fibroblasts and tenocytes from *Flii*^*+/−*^, WT, and *Flii*^*Tg/Tg*^ mice were left for 48 h in a 2D culture to allow the accumulation of secreted collagen type III. **p**–**r** There was no difference seen in collagen type III secretion in tenocytes across all three genotypes. **s**–**u** Representative images show that overexpression of *Flii* significantly increased the level of collagen type III secretion in fibroblasts. Magnification × 10, scale bar = 200 μM. **v** Graphical representation of collagen type III secretion in fibroblasts and tenocytes. Data is represented as mean ± SEM. *p ≤ 0.05, **p ≤ 0.01. *n* = 6

*Flii*^*+/−*^, WT, and *Flii*^*Tg/Tg*^ tenocytes and fibroblasts were incorporated into a 3D floating collagen gel matrix and left for 72 h to assess the rate of contraction. Minimal contraction was seen in the tenocyte gels across the 72 h with no significant difference seen between the genotypes (Fig. 6a–c, g). In contrast, significant contraction was seen in the fibroblast gels across the 72 h, with *Flii*^*+/−*^ gels showing significantly increased contraction than WT and *Flii*^*Tg/Tg*^ gels at 48 h (*p* = 0.004 and 0.0001, respectively) and *Flii*^*Tg/Tg*^ at 72 h (*p* = 0.00007) (Fig. 6d–f, h).

### Flii affects collagen type I and collagen type III production in unwounded fibroblasts but not tenocytes

Collagen type I is the main component of tendons, providing stability and strength. Here the effect of *Flii* on collagen type I and III production in tenocytes and fibroblasts from *Flii*^*+/−*^, WT, and *Flii*^*Tg/Tg*^ mice was investigated. No significant difference was observed in collagen type I production in tenocytes with different levels of Flii; however, fibroblasts were observed to have secreted significantly more collagen type I than the tenocytes (Fig. 6i–o). Overexpression of *Flii* in *Flii*^*Tg/Tg*^ fibroblasts significantly decreased collagen type I production when compared with fibroblasts from WT and *Flii*^*+/−*^ mice (*p* = 0.0002 and 0.03, respectively) (Fig. 6l–o). Similarly, no significant difference in collagen type III production was observed in the tenocytes across all three genotypes (Fig. 6p–r, v); however, fibroblasts showed an overall significant increase in collagen type III production (Fig. 6s–v) Furthermore, *Flii*^*Tg/Tg*^ fibroblasts showed significantly increased collagen type III secretion when compared to fibroblasts from WT and *Flii*^*+/−*^ mice (*p* = 0.0002 and 0.003, respectively) (Fig. 6v).

### Collagen type I production is delayed in scratch-wounded *Flii*^*Tg/Tg*^ tenocytes

**Fig. 7:**
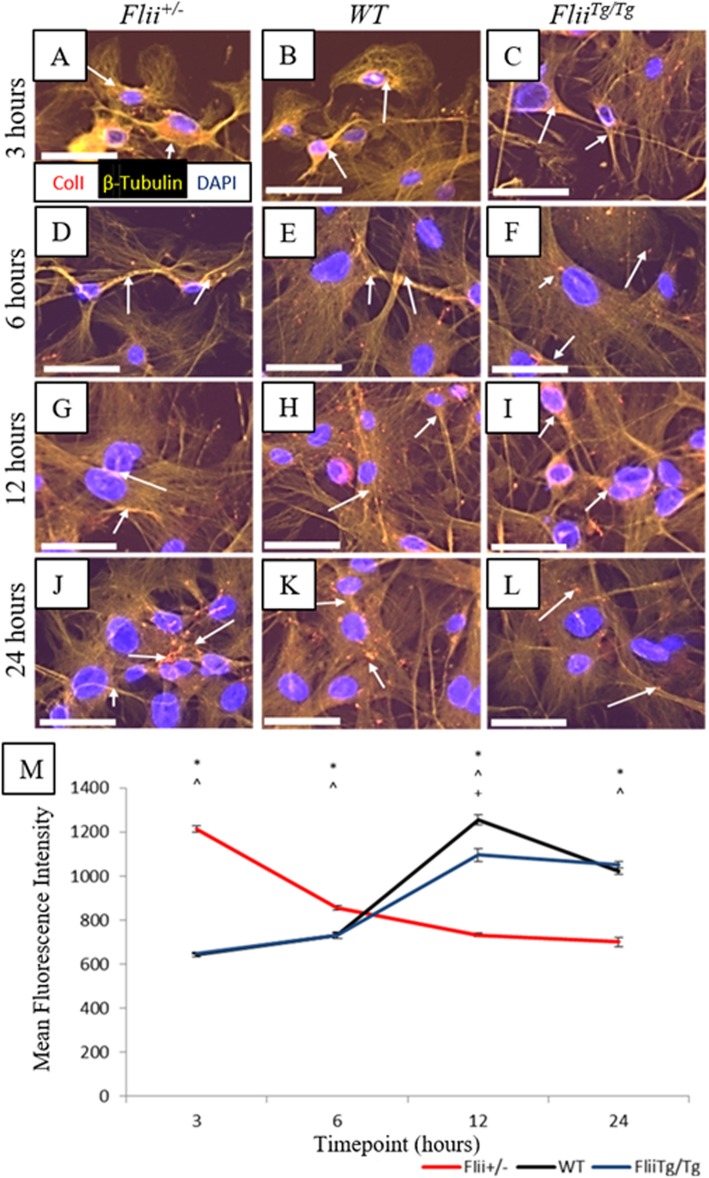
*Flii*^*Tg/Tg*^ tenocytes show decreased collagen type I expression in response to wounding in vitro. Confluent tenocytes were scratch-wounded and stained for collagen type I expression at 3, 6, 12, and 24 h post-wounding. **a**–**l** Representative images showing collagen type I expression in *Flii*^*+/−*^, WT, and *Flii*^*Tg/Tg*^ tenocytes at 3, 6, 12, and 24 h post-wounding. Magnification × 10. Scale bar = 100 μM. Collagen type I represented by white arrows. **m** Graphical representation of collagen type I expression in tenocytes. *Flii*^*Tg/Tg*^ tenocytes show a later peak of collagen type I release than *Flii*^*+/−*^ cells. *p ≤ 0.05 between *Flii*^*+/−*^ and *Flii*^*Tg/Tg*^. ^^^*p* ≤ 0.05 between *Flii*^*+/−*^ and WT. ^+^*p* ≤ 0.05 between *Flii*^*Tg/Tg*^ and WT. Data represented as mean ± SEM. *n* = 6

Previous studies have shown that Flii can regulate collagen I production during cutaneous wound healing [8]. Here we examined the effect of differential Flii expression on collagen type I production by wounded tenocytes using established collagen I assay [27]. Tenocytes from *Flii*^*+/−*^, WT, and *Flii*^*Tg/Tg*^ mice were scratch-wounded and immunofluorescently stained for collagen type I expression at 3, 6, 12, and 24 h post-wounding (Fig. 7a–l). *Flii*^*Tg/Tg*^ tenocytes had a significant delay in peak collagen type I expression at 12 h post-wounding compared to *Flii*^*+/−*^collagen type I expression at 3 h post-wounding; however, the peak level of collagen type I expression was not significantly different (Fig. 7a–m). Collagen type I expression had returned to basal levels in the *Flii*^*+/−*^ tenocytes by 12 h and remained low for the rest of the experiment whereas there was still elevated expression in the *Flii*^*Tg/Tg*^ tenocytes by 24 h (Fig. 7m).

### *Flii*^*Tg/Tg*^ tenocytes express significantly less TGFβ1 during healing

**Fig. 8:**
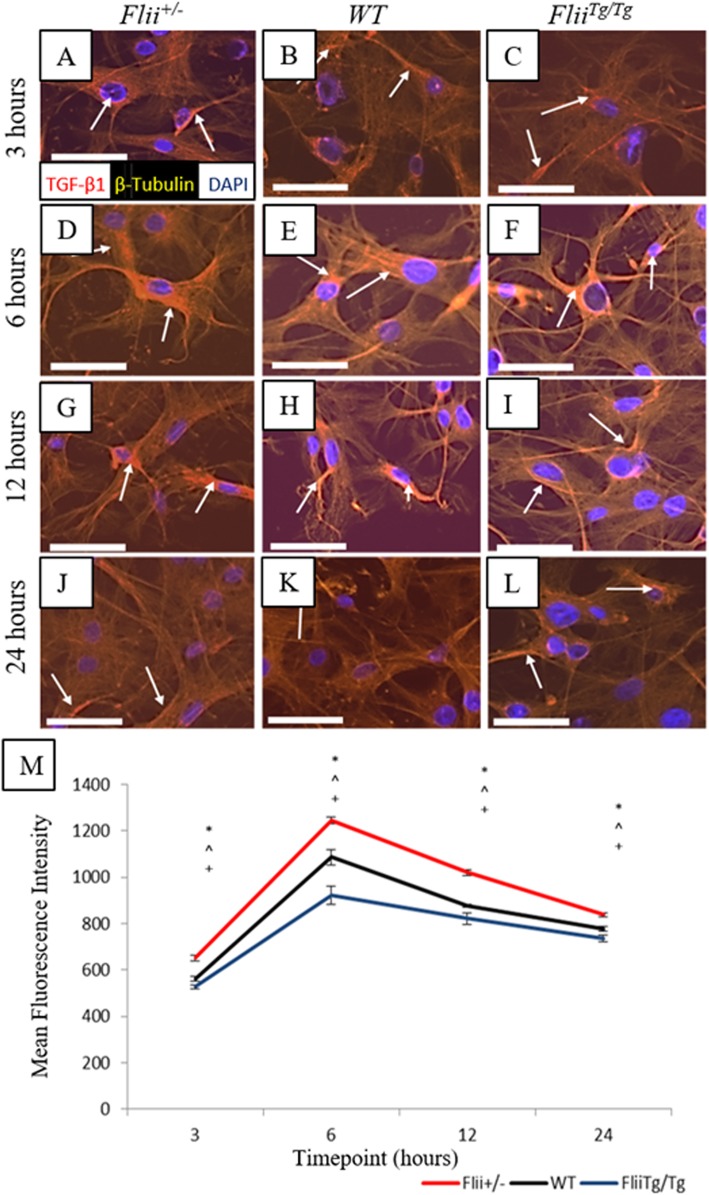
*Flii*^*Tg/Tg*^ tenocytes express significantly less TGFβ1 than WT or *Flii*^*+/−*^ tenocytes following scratch wounding in vitro. Confluent tenocytes were scratch-wounded and stained for TGFβ1 expression at 3, 6, 12, and 24 h post-wounding. **a**–**l** Representative images showing TGFβ1 expression in *Flii*^*+/−*^, WT, and *Flii*^*Tg/Tg*^ tenocytes at 3, 6, 12, and 24 h post-wounding. Magnification × 10. Scale bar = 100 μM. TGFβ1 represented by white arrows. **m** Graphical representation of TGFβ1 expression in tenocytes. TGFβ1 expression is significantly decreased in *Flii*^*TgTg*^ tenocytes compared to WT and *Flii*^*+/−*^ cells. *p ≤ 0.05 between *Flii*^*+/−*^ and *Flii*^*Tg/Tg*^. ^^^*p* ≤ 0.05 between *Flii*^*+/−*^ and WT. ^+^*p* ≤ 0.05 between *Flii*^*Tg/Tg*^ and WT. Data represented as mean ± SEM. *n* = 6

TGFβ1 is often referred to as the pro-scarring growth factor in the TGFβ family, and increased levels of this growth factor are generally associated with poorer healing outcomes and more significant scarring and fibrosis [30]. Previous studies have described the effect of differential Flii levels on TGF-β expression during wound healing [31]. Here we examined the effect if differential Flii levels on tenocyte TGF-β1 expression. Tenocytes from *Flii*^*+/−*^, WT, and *Flii*^*Tg/Tg*^ mice were scratch-wounded and immunofluorescently stained for TGFβ1 expression at 3, 6, 12, and 24 h post-wounding (Fig. 8a–l). TGFβ1 expression was significantly decreased across all time points in *Flii*^*Tg/Tg*^ tenocytes compared to WT (*p* = 0.03, 0.0005, 0.003, and 0.01, respectively) and *Flii*^*+/−*^ tenocytes (*p* = 0.0003, 0.00003, 0.00007, and 0.00004, respectively) (Fig. 8a–m). Tenocytes showed a significant increase in expression between 3–6 h, suggesting secretion of TGFβ1 occurs early in the healing process, with a gradual decline in expression over the remainder of the experiment (Fig. 8m).

## Discussion

Tendon injuries represent a multifaceted model of cellular change, requiring a complex re-organization of the actin cytoskeleton in order to facilitate essential processes for healing including migration, proliferation, contraction, and secretion. Tenocytes make up the majority of the cellular content in tendons and are integral in coordinating the healing process following injury [32]. Tenocytes are responsible for releasing signaling molecules to recruit a variety of growth factors, cytokines, and inflammatory cells to the injury site in order to facilitate successful healing [32]. It is well established that both tenocytes and dermal fibroblasts are of mesodermal origin, and both cell types exhibit similar spindle-shaped morphology when grown in in vitro culture. Both produce major extracellular matrix proteins and growth factors in response to injury. For these reasons, tenocyte function is often compared to dermal fibroblasts; however, it is important to note that they are distinct cell types. Tenocytes are mechanosensitive cells with unique properties that detect and respond to changes in mechanical load via deformation of their cell membrane and actin cytoskeleton [33]. Our findings that tenocytes and fibroblasts exhibit different cellular responses to changes in *Flii* gene expression aligns with previous studies that have shown tenocytes behave differently to dermal fibroblasts in vitro [34] as well as previous studies that show *Flii* positively affects digit regeneration and rodent vibrissa healing [35, 36].

Previous studies have shown that the upregulation of Flii is associated with impaired migration, proliferation, and adhesion of both fibroblasts and keratinocytes [8, 17]. Flii has previously been reported to act as an intracellular protein that affects actin remodeling and cytoskeleton organization through association with different signaling proteins [8, 11, 13]. Importantly, Flii translocates from the cytoplasm to the nucleus in response to wounding in fibroblasts but not keratinocytes. This suggests that the cell-specific nuclear translocation of Flii may directly regulate gene expression in fibroblasts but not keratinocytes, providing a potential mechanism of action for Flii in wound healing [31]. Using an inducible ROSA26 system to conditionally over-express Flii in fibroblasts resulted in impaired healing responses suggesting that fibroblast-specific production of Flii contributes to wound impaired healing [37]. In this study, increased Flii expression in tenocytes led to an improvement in tenocyte migration, proliferation, and outgrowth, providing more support for the important cell-specific and cell-type-dependent functions of Flii.

Cellular migration is an essential part of the healing process, allowing cells to move across a space created by an injury, to re-establish continuity and strength to an injured area. In order for cells to migrate, a complex cascade of events occurs, with actin assembly driving lamellipodia and filopodia forward, and adhesions forming to anchor the cell body to the surface [9]. Rear adhesions disassemble to allow the cell to retract its rear edge and the cell to move forward. Actin reassembly is integral to this process and actin remodeling proteins such as Flii are essential to ensure this progression occurs correctly [9]. *Flii*^*Tg/Tg*^ tenocytes showed significantly improved migration and proliferation over 24 h compared with WT and *Flii*^*+/−*^ counterparts, indicating the important role of Flii in regulating tenocyte adhesion, migration, and proliferation. These findings are in agreement with previous studies showing Flii association with different signaling proteins involved in actin rearrangement including Cdc42 and the Ras-dependent adenylyl cyclase pathway [15, 38]. Additionally, studies have shown that Flii regulates focal adhesion turn-over and cell migration by inhibition of paxillin phosphorylation via a Rac-1-dependent pathway [13, 39] and its ability to cap actin filaments [40]. Experiments presented in this manuscript show the effects of Flii on tenocyte cell function, further supporting the role of Flii in regulating cellular responses during homeostasis and in response to wounding.

Fibroblasts and tenocytes are both of mesodermal origin and both display similar morphology in vitro as well as being producers of major extracellular proteins such as collagen [41]. Collagen type I is the main component of tendons and tendon adhesions and is an essential protein synthesized during the healing process, providing structure and tensile strength. No significant difference in collagen type I production was observed in the tenocytes while fibroblasts secreted significantly more collagen type I. Overexpression of *Flii* in *Flii*^*Tg/Tg*^ fibroblasts significantly decreased collagen type I production when compared to fibroblasts from WT and *Flii*^*+/−*^ mice. Again, there was no significant difference in collagen type III secretion seen between tenocytes across all three genotypes while fibroblasts showed an overall significant increase in collagen type III production. Flii does have an effect on both collagen type I and collagen type III production as seen in fibroblasts, both in this study and previously [8]. It is, however, the lack of collagen production seen in tenocytes suggest that these cells are less metabolically active than fibroblasts during the “stationary phase” of cell mitosis; this is supported by Stoll et al. that showed slow proliferation and unstable tenocyte phenotype in monolayer cultures [42]. In response to wounding, collagen production in tenocytes is increased and the ratio of collagen type I and collagen type III is also increased [43]. One limitation of this study is the lack of in vivo studies and future in vivo studies in Flii genetic mice will provide more information about the potential effect of differential Flii levels on collagen type I and collagen type III production and organization during the healing of tendon injuries. ECM disorganization is known as a major hallmark in tendinopathy when a significant decrease in type I collagen is prevalent [43].

No significant difference was observed between *Flii*^*+/−*^, WT, and *Flii*^*Tg/Tg*^ tenocytes in a 3D collagen gel contraction model suggesting that *Flii* does not affect tenocyte contraction. Interestingly, this was opposite to previous observations on the effect of Flii on fibroblast collagen gel contraction [16]. Embryonic tenocytes have actin-rich fibripositors which act via contraction in order to form the “crimp” structure seen in established tendons. This structure is vital for the mechanical shock-absorbing behavior of mature tendons [44, 45]. Adult tendons show minimal contraction during healing and the “crimp” structure tends to be lost in the re-established tendon after healing is complete. So, although *Flii* appears to have minimal effect on adult tenocyte contraction in vitro, *Flii* is an important developmental regulator [8, 21] and has been shown to be involved in fibroblast contraction [16], and so further investigations into its role in embryonic tenocyte cell contraction should be examined.

Many studies have shown that TGFβ1 is a regulator of fibrosis, and excess TGFβ1 levels have been associated with poorer healing outcomes and excessive scarring [46, 47]. This is associated with the inflammatory phase of healing and prolonged expression of TGFβ1 can lead to an extension of the inflammatory phase which could contribute towards poorer healing outcomes. In a cutaneous burn injury model, elevated Flii leads to increased TGFβ gene expression and reduced Flii levels are linked with decreased TGFβ1 expression leading to better healing outcomes [48]. To investigate the possible mechanisms behind which overexpression of *Flii* in tenocytes may affect collagen deposition, the expression of TGFβ1 in response to in vitro scratch wounding of tenocytes was investigated. *Flii*^*Tg/Tg*^ tenocytes expressed significantly less TGFβ1 compared with their WT and *Flii*^*+/−*^ counterparts. TGFβ1 has been shown to increase collagen type I production resulting in excessive and disordered organization, leading to increased tendon adhesion size and severity [7]. *Flii*^*Tg/Tg*^ tenocytes expressed significantly lower TGFβ1 levels during migration, and at 12 h post-scratch wounding, these cells had significantly lower collagen type I levels than their WT and *Flii*^*+/−*^ counterparts. Decreased levels of pro-scarring TGFβ1 coupled with decreased and more controlled production of type I collagen, by *Flii*^*Tg/Tg*^ tenocytes, suggest a potential positive role for Flii on healing of tendon injuries.

## Conclusions

Fibroblast behavior has been extensively studied in cutaneous healing experiments, and a reduction in *Flii* results in improved fibroblast migration, proliferation, adhesion, and contraction [8, 16]. The opposite is observed in tenocytes in this study suggesting cell-specific effects of Flii may occur in response to different stimuli. This study clearly suggests a positive role for Flii in regulating tenocyte proliferation and migration. In conjunction with decreased collagen type I and TGF-β production post wounding, this identifies Flii as a novel target for modulating tenocyte activity and for improving tendon repair. While the exact mechanism of Flii effects on tendon repair is still to be determined, the outcome of this study may help to advance new therapeutic approaches aimed at improving the healing of tendon injuries.

## Data Availability

The datasets used and/or analyzed during the current study are available from the corresponding author on reasonable request.

## Abbreviations

DAPI: 4′,6-Diamindino-2-phenylindole
ECM: Cell-extracellular matrix
Flii: Flightless
LRR: Leucine-rich repeat
MTJ: Myotendinous junction
TGF-β: Transforming growth factor beta
WT: Wild-type
